# Lignocellulosic saccharification by a newly isolated bacterium, *Ruminiclostridium thermocellum* M3 and cellular cellulase activities for high ratio of glucose to cellobiose

**DOI:** 10.1186/s13068-016-0585-z

**Published:** 2016-08-11

**Authors:** Tao Sheng, Lei Zhao, Ling-Fang Gao, Wen-Zong Liu, Min-Hua Cui, Ze-Chong Guo, Xiao-Dan Ma, Shih-Hsin Ho, Ai-Jie Wang

**Affiliations:** 1 State Key Lab of Urban Water Resource and Environment, Harbin Institute of Technology, Harbin, 150090 China; 2 CAS Key Laboratory of Environmental Biotechnology, Research Center for Eco-Environmental Sciences, Chinese Academy of Sciences, Beijing, China; 3 Advanced Water Management Centre, Faculty of Engineering, Architecture and Information Technology, The University of Queensland, Brisbane, QLD 4072 Australia

**Keywords:** Cellulase, Glucose, Lignocellulose, Oligosaccharides, *Ruminiclostridium thermocellum*, Saccharification

## Abstract

**Background:**

Lignocellulosic biomass is one of earth’s most abundant resources, and it has great potential for biofuel production because it is renewable and has carbon-neutral characteristics. Lignocellulose is mainly composed of carbohydrate polymers (cellulose and hemicellulose), which contain approximately 75 % fermentable sugars for biofuel fermentation. However, saccharification by cellulases is always the main bottleneck for commercialization. Compared with the enzyme systems of fungi, bacteria have evolved distinct systems to directly degrade lignocellulose. However, most reported bacterial saccharification is not efficient enough without help from additional β-glucosidases. Thus, to enhance the economic feasibility of using lignocellulosic biomass for biofuel production, it will be extremely important to develop a novel bacterial saccharification system that does not require the addition of β-glucosidases.

**Results:**

In this study, a new thermophilic bacterium named *Ruminiclostridium thermocellum* M3, which could directly saccharify lignocellulosic biomass, was isolated from horse manure. The results showed that *R. thermocellum* M3 can grow at 60 °C on a variety of carbon polymers, including microcrystalline cellulose, filter paper, and xylan. Upon utilization of these substrates, *R. thermocellum* M3 achieved an oligosaccharide yield of 481.5 ± 16.0 mg/g Avicel, and a cellular β-glucosidase activity of up to 0.38 U/mL, which is accompanied by a high proportion (approximately 97 %) of glucose during the saccharification. *R. thermocellum* M3 also showed potential in degrading natural lignocellulosic biomass, without additional pretreatment, to oligosaccharides, and the oligosaccharide yields using poplar sawdust, corn cobs, rice straw, and cornstalks were 52.7 ± 2.77, 77.8 ± 5.9, 89.4 ± 9.3, and 107.8 ± 5.88 mg/g, respectively.

**Conclusions:**

The newly isolated strain *R. thermocellum* M3 degraded lignocellulose and accumulated oligosaccharides. *R. thermocellum* M3 saccharified lignocellulosic feedstock without the need to add β-glucosidases or control the pH, and the high proportion of glucose production distinguishes it from all other known monocultures of cellulolytic bacteria. *R. thermocellum* M3 is a potential candidate for lignocellulose saccharification, and it is a valuable choice for the refinement of bioproducts.

**Electronic supplementary material:**

The online version of this article (doi:10.1186/s13068-016-0585-z) contains supplementary material, which is available to authorized users.

## Background

Lignocellulosic biomass is one of the most abundant resources on earth. However, until now, most lignocellulosic wastes are either unutilized or combusted directly [1], which causes serious environmental pollution. To reclaim wastes and mitigate the world’s dependence on fossil fuels, the bioconversion of lignocellulosic biomass to biofuel has drawn increasing attention during the past decade [2–4]. However, with respect to lignocellulosic biomass conversion, current technologies are still not suitable for large-scale applications because of the crystallinity and heterogeneity of feedstocks. Pretreatment, one of the main bottlenecks [5], is first required to separate celluloses from the lignin, and then the newly freed carbohydrate polymers should be hydrolyzed to simple monosaccharides. Lignocellulose saccharification is conventionally accomplished through physical, chemical, and biological methods [6]. Although physical and chemical saccharification can effectively hydrolyze lignocellulose, huge amounts of toxic derivatives of bran aldehydes, anthracene, and furfural are generated by these processes [7], which significantly inhibits subsequent fermentation [8]. Moreover, physical and chemical saccharification usually proceed under severe reaction conditions; thus, corrosion-resistant and high-pressure reactors are required, which will markedly increase the reaction costs [9]. Conversely, biological saccharification can be performed under mild conditions to hydrolyze lignocellulose effectively without producing downstream derivatives; thus, it is regarded as an ideal process for lignocellulose saccharification [10].

Biological saccharification of lignocellulosic biomass in nature is generally considered to be performed by a variety of microorganisms or microbial communities, including fungi and bacteria. To date, most biological processes employ cellulases secreted by wild-type fungi, such as *Trichoderma reesei* [11], *Fusarium oxysporum* [12], *Piptoporus betulinus* [13], *Penicillium echinulatum* [14], *Penicillium purpurogenum* [15], *Aspergillus niger* [16], and *Aspergillus fumigatus* [17], for saccharification. However, in terms of the specific cellulolytic activity, the cellulase system of the thermophilic, anaerobic bacterium *Clostridium thermocellum* has been reported to degrade cellulose more effectively than fungal enzyme systems [18]. Additionally, several anaerobic bacteria, such as *Caldicellulosiruptor saccharolyticus* [19], *Caldicellulosiruptor lactoaceticus* [20], *Ruminococcus albus* [21], and *Clostridium cellulofermentans* [22], which have evolved distinct enzyme systems [23], were reported to efficiently and directly degrade lignocellulose. Using multi-enzyme complexes, these bacteria can saccharify lignocellulose during cultivation, which would greatly reduce operating costs [24, 25].

These anaerobic, lignocellulose-degrading microbes usually reside in the digestive systems of some cellulose-feeding animals, such as cows, horses, and termites, which can effectively utilize cellulose substrates as their main foods. A recent study showed that bacteria in the hindgut of horses could effectively convert a high-fiber, forage-based feedstock into small molecules [26]. Thus, the examination of this natural biomass utilization system may have great potential to identify mechanisms, enzymes, and organisms for further improving managed industrial processes for biomass conversion. However, it is difficult to simulate the environment of the hindgut when growing bacteria. Recently, it was reported that the dominant phyla in horse manure were mainly *Firmicutes, Proteobacteria, Verrucomicrobia*, *Bacteroidetes*, and *Ruminococcus*, which is similar to the microbial community structure in the hindgut of horses [27]. In this regard, characterizing some individual microorganisms isolated from horse manure may be a promising method to obtain a unique consortium of microorganisms that are capable of effectively degrading lignocellulosic biomass.

Although lignocellulose-degrading bacteria have several advantages in lignocellulosic degradation, the sources of cellulosic bacteria, especially cellulosic bacteria that can accumulate oligosaccharides or produce high-value products, are rare. Such bacteria would be valuable for pilot- or commercial-scale applications. Therefore, it is necessary to identify new sources of these bacteria and investigate their ability to saccharify lignocellulosic biomass.

## Results

### Enrichment of cellulose-degrading thermophilic bacteria for oligosaccharide accumulation

**Fig. 1:**
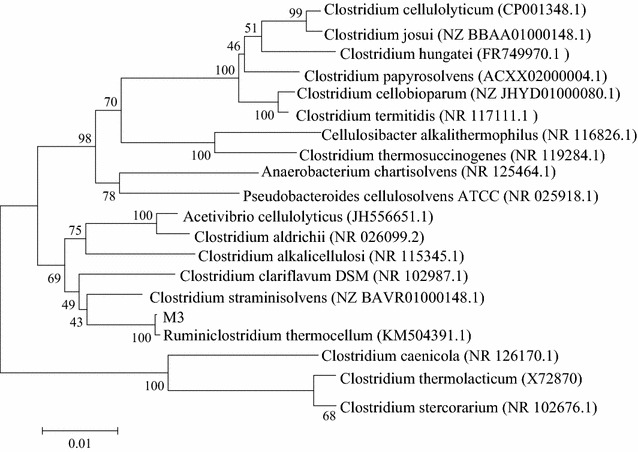
Phylogenetic relationship of *R. thermocellum* M3 and other strains based on 16S rDNA gene sequences. Numbers along branches indicate bootstrap values with 1000 times

The results showed that a culture enriched from horse manure degraded Avicel and accumulated oligosaccharides (Additional file 1). A microbial community analysis showed that the bacterial community of the horse manure was similar throughout the enrichment process (Additional file 2). According to sequencing and BLAST analyses, *Ruminiclostridium thermocellum* was the predominant specie. To isolate functional organisms, a serially diluted, stable, enriched culture was plated on cellulose agar, and bacterial colonies with extensive clearing zones were screened. One isolated strain was capable of producing oligosaccharides from cellulose. An analysis of the 16S rDNA gene sequence of this strain indicated that it is a member of the genus *Ruminiclostridium*. A phylogenetic analysis of 16S rDNA genes revealed that the strain shared 99 % sequence identity with the 16S rDNA gene of *R. thermocellum* ATCC 27045 (Fig. 1; Additional file 3). Therefore, this strain was identified and named *R. thermocellum* M3. The results of series of physiological and biochemical experiments showed that *R. therm*o*cellum* M3 is an oval-shaped (0.5–0.7 × 2.0–3.0 μm), Gram-positive bacterium with no visible tufted flagella, as shown in Additional file 4. Table 1 shows that *R. thermocellum* M3 utilized a wide range of carbon sources such as glucose, fructose, Avicel, and carboxymethyl cellulose. Organic nitrogen sources, such as yeast extract and beef extract, and even inorganic nitrogen sources such as (NH_4_)_2_SO_4_, NH_4_Cl, and NaNO_3_, served as nitrogen sources for *R. therm*o*cellum* M3 (Table 1). The main metabolic products of *R. thermocellum* M3 were acetate, along with lower amounts of ethanol and lactate; moreover, butyrate and butanol were not observed.

**Table 1:** Physiological properties of *R. thermocellum* M3

Characteristic	Value	Substrate utilization	Value
Gram staining	+	Avicel	+
		Glucose	+
		Fructose	+
		Xylose	−
		Maltose	+
		Lactose	−
Morphology	Short rod-shaped, spore	Sucrose	+
Anaerobic growth	+	Cellobiose	+
Motility	+	Xylitol	−
Sulfate reduction	−	CMC	+
Nitrate reduction	−	Starch	+
Gelatin hydrolysis	+		
Metabolic products with cellulose	Acetate, ethanol, lactate	Filter paper	+
		NaCl tolerance	1.25 %
		Beef extract	+
		(NH_4_)_2_SO_4_	+
		NH_4_Cl	+
		NaNO_3_	+

− negative; + positive

### Fermentation during defined cultivation conditions

**Fig. 2:**
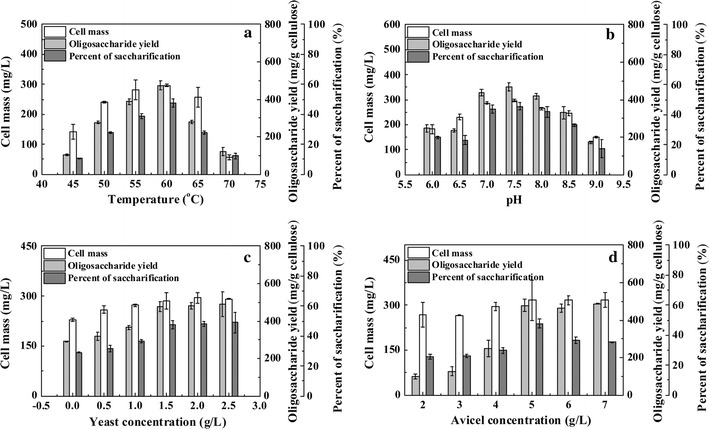
Fermentation profile of *R. thermocellum* M3 under different cultivation conditions. **a** Effects of temperature on Avicel saccharification. **b** Effects of pH on Avicel saccharification. **c** Effects of YE concentration on Avicel saccharification. **d** Effects of Avicel concentration on Avicel saccharification

*Ruminiclostridium thermocellum* M3 exhibited cellulose saccharification ability over a wide temperature range, from 45 to 70 °C, as shown in Fig. 2a. The oligosaccharide yield increased from 105.6 ± 14.2 to 474.4 ± 25.9 mg/g Avicel as the temperature increased from 45 to 60 °C; however, the yield decreased when the temperature was further increased to 70 °C. The profile of the cell mass concentration was similar to the oligosaccharide yield (Fig. 2a). The maximum cell mass concentration of 295.9 ± 13.7 mg/L was obtained at 60 °C. In addition, as depicted in Fig. 2b, *R. thermocellum* M3 saccharified Avicel at pHs ranging from 6.0 to 9.0. The optimal pH for Avicel saccharification and cell growth was 7.5, and the maximum values reached 468.8 ± 21.5 mg/g Avicel and 296.8 ± 4.7 mg/L, respectively.

Yeast extract (YE) is one of the most important nitrogen sources for bacteria [28]. Figure 2c shows that the oligosaccharide yield increased when the YE concentration increased from 0 to 2.5 g/L. A similar profile was observed for the cell mass. The cell mass increased from 229.5 ± 5.1 to 291.4 ± 4.2 mg/L as the YE concentration increased from 0 to 2.5 g/L. However, Avicel saccharification remained constant when the YE concentration exceeded 1.5 g/L. Approximately 48 % of the Avicel was saccharified at a YE concentration of 1.5 g/L.

We also investigated the optimal substrate concentration for oligosaccharide production. Figure 2d shows that *R. thermocellum* M3 grew on Avicel at concentrations ranging from 2.0 to 7.0 g/L, and that the cell mass concentration increased from 268.2 ± 32.2 to 317.4 ± 25.7 mg/L, respectively. At a concentration of 2.0 g/L, approximately 25 % of the Avicel could be saccharified, and this value increased to 47.8 ± 3.2 % when the Avicel concentration increased to 5.0 g/L; however, the saccharification proportion decreased to 34.80 ± 0.35 % as the Avicel concentration increased to 7.0 g/L. Unlike the cell mass and the saccharification proportion, the oligosaccharide yield gradually increased as the Avicel concentration increased from 2.0 to 5.0 g/L, and then it remained constant as the concentration increased from 5.0 to 7.0 g/L.

### Avicel biodegradation characteristics

**Fig. 3:**
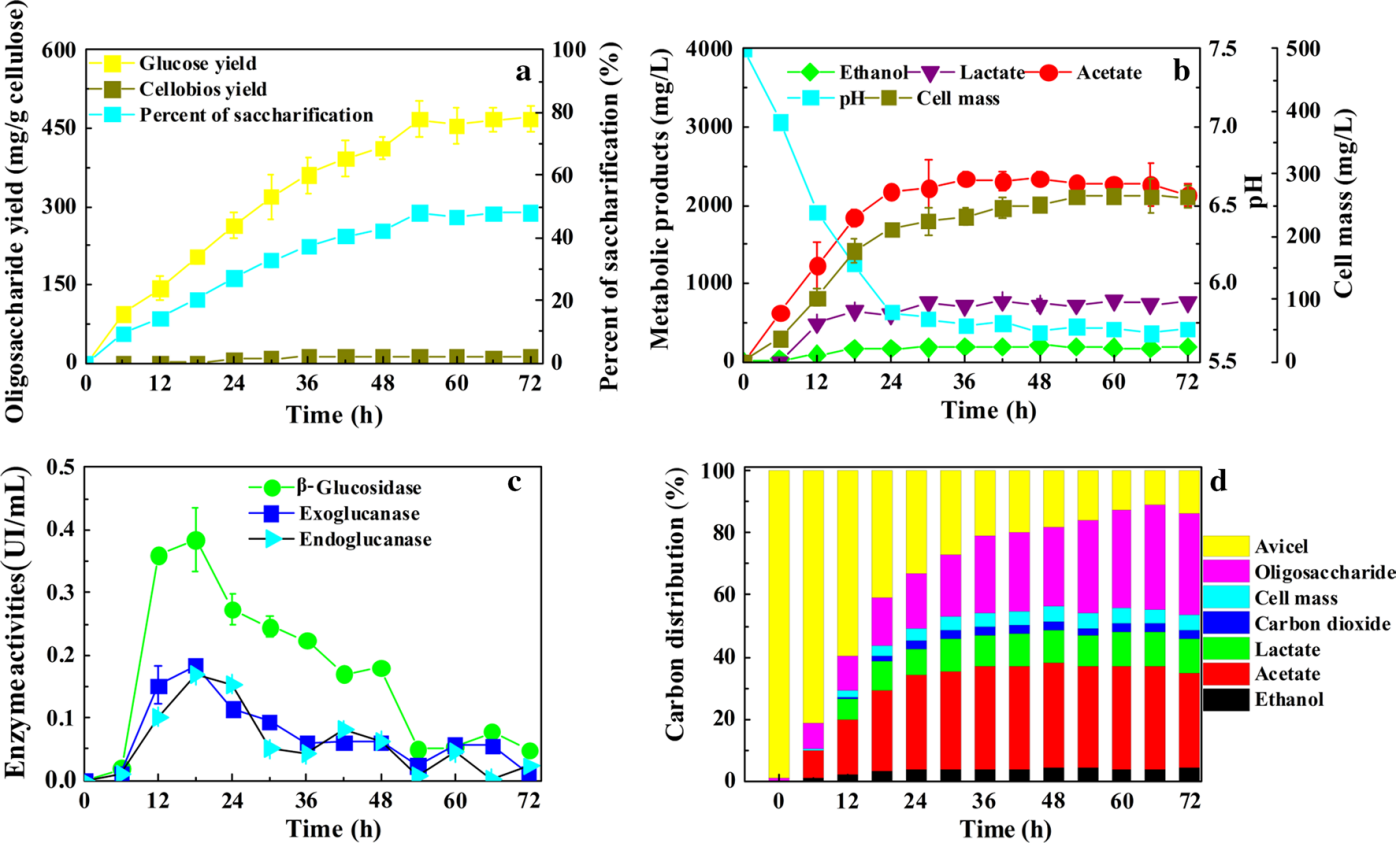
The time course profiles and kinetics of batch fermentation in 5 g/L Avicel medium. **a** Cellulose and oligosaccharides. **b** Metabolites, cell mass concentration and pH profile. **c** Carbon balance. **d** Enzyme activities

To investigate the cellulose saccharification kinetics of *R. thermocellum* M3 under optimal conditions, *R. thermocellum* M3 was inoculated in optimized ATCC 1191 medium (MA medium) (Avicel 5 g/L, YE 1.5 g/L) at 60 °C, pH 7.5. As shown in Fig. 3a, during the fermentation process, the Avicel concentration decreased gradually; approximately 47 % of the Avicel was saccharified after 54 h of fermentation. The oligosaccharide yield simultaneously increased with increasing cell mass, reached its maximum at 54 h, and then remained constant. Glucose accounted for the majority (approximately 97 %) of the products (466.7 ± 34.8 mg/g Avicel), and it was accompanied by a small amount of cellobiose (<3 %). Cellobiose was barely observed before 18 h; however, the cellobiose concentration increased to 12.0 ± 0.5 mg/g Avicel at 30 h and remained at 12 mg/g Avicel until the end of the fermentation. As incubated to the fresh medium, the concentrations of the metabolites (e.g., acetate, lactate) gradually increased. After 54 h of fermentation, the acetate and lactate concentrations increased to 2300 and 700 mg/L, respectively. Meanwhile, the pH decreased to below 6.0 (Fig. 3b). Similar to the metabolic products, the cell mass concentration also peaked at 263.3 ± 18.6 mg/L at 54 h and then remained constant.

To confirm that a pH decrease causes the oligosaccharide accumulation to plateau during fermentation, glucose was also used as a substrate at an initial pH of 7.5 and 60 °C. When glucose was selected as the carbon source, the glucose concentration was consistent with the trend of pH and cell mass when the pH approached 5.5. At this pH, little cell growth was observed, and the glucose concentration remained constant (Additional file 5).

The carbon balance of *R. thermocellum* M3 was further evaluated based on the utilization of Avicel and the production of cell biomass (total protein), oligosaccharides, and end products, and carbon closures ranged from 99.6 to 96.8 %. Initially, carbon balance closures of 99.6 ± 0.3 % were observed at 6 h. At this point, approximately 0.6 and 8 % of the carbon flowed into the cell mass and oligosaccharides, respectively. After 24 h, the value increased to 4.3 % (cell mass) and 17.9 % (oligosaccharides). When the cultivation increased to 72 h, the weights of the cell mass and metabolites remained constant; however, the oligosaccharides increased from 17.9 to 32.4 %, and the carbon balance closure declined slightly to 96.8 ± 0.2 %. This indicated that volatile acids and oligosaccharides were the dominant components in the end stages. Approximately 46 % of the total carbon was converted to acetate, lactate, and ethanol, and approximately 33 % of the total carbon was converted to oligosaccharides; the rest of the carbon was used for the production of CO_2_ and cell mass (Fig. 3c).

The cell-associated cellulase activities of *R. thermocellum* M3 under the optimal culture conditions were also determined, and they are shown in Fig. 3d. The cell-associated cellulase activities increased gradually within 18 h of fermentation and decline rapidly thereafter. The main type of cellulase was β-glucosidase (maximum cellulase activity of approximately 0.38 U/mL), followed by exoglucanase (maximum cellulase activity of approximately 0.18 U/mL) and endoglucanase (maximum cellulase activity of approximately 0.16 U/mL). Different from the activities of the cell-associated cellulases, the activities of extracellular cellulases in the culture supernatant increased continuously until 24 h of culture (Additional file 6). The main type of extracellular cellulase was β-glucosidase (maximum cellulase activity of approximately 0.35 U/mL), followed by exoglucanase (maximum cellulase activity of approximately 0.18 U/mL) and endoglucanase (maximum cellulase activity of approximately 0.17 U/mL). The peak cellulase activities of *R. thermocellum* M3 were obtained before 28 h. The cell-associated cellulase specific activities were slightly higher than the extracellular cellulase activities (Additional file 7).

### Biodegradation characteristics of untreated lignocellulosic biomass

**Fig. 4:**
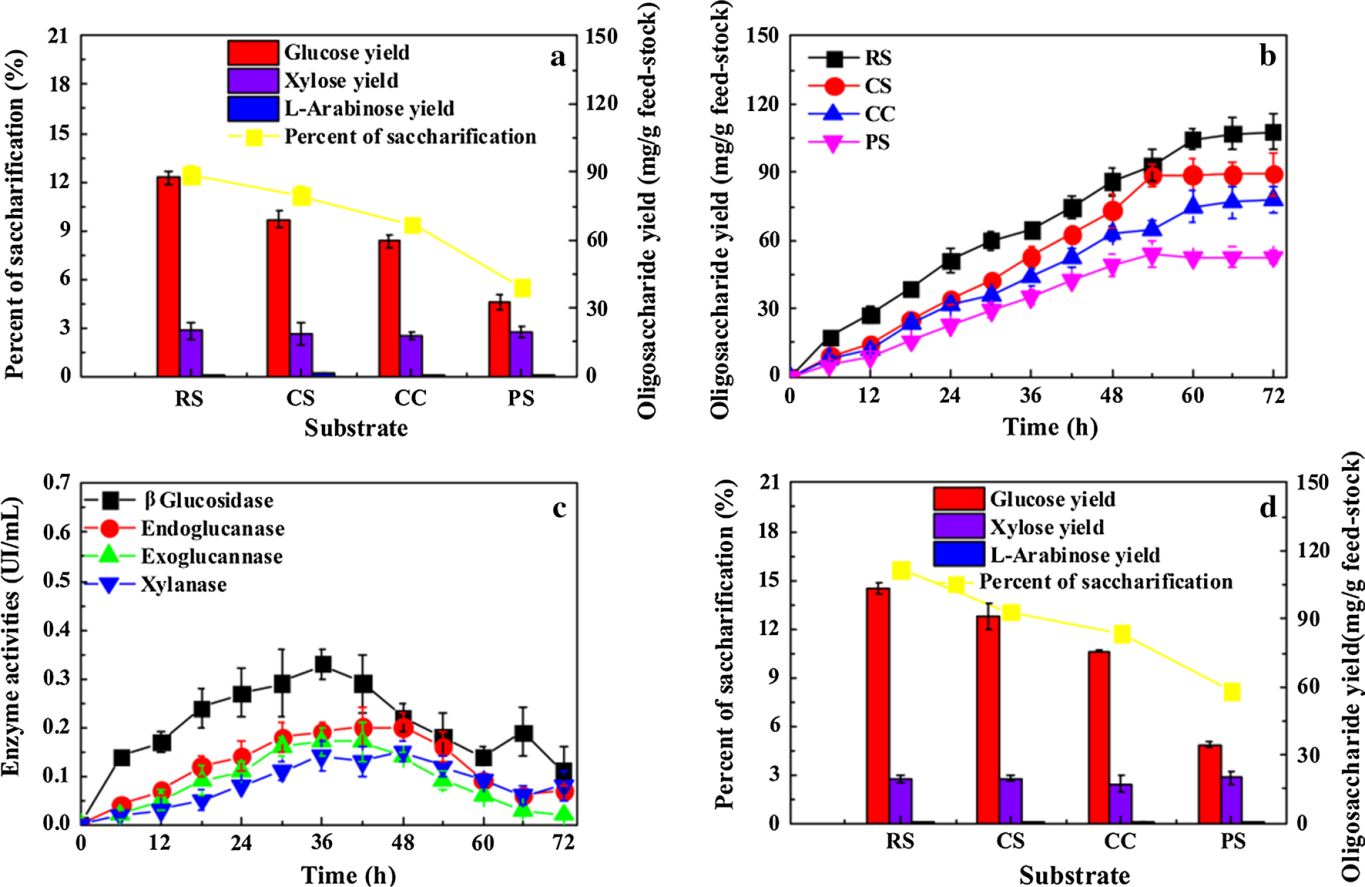
The time course profiles and kinetics of batch fermentation in 5 g/L untreated lignocellulose medium. **a** Oligosaccharide composition. **b** Oligosaccharide yield. **c** Enzyme activities. **d** Saccharification by commercial cellulase

To assess the ability of *R. thermocellum* M3 to convert lignocelluloses into oligosaccharides, natural lignocellulosic biomass (e.g., rice straw, corn straw, corn cobs, and poplar sawdust) were selected as substrates. In general, oligosaccharides could be generated from all kinds of substrates, as shown in Fig. 4a. The lignocellulosic biomass hydrolysate mainly consisted of glucose and xylose (Fig. 4a), and the oligosaccharide yields varied with the different substrates as follows: rice straw (107.8 ± 7.7 mg/g) > corn straw (89.4 ± 9.3 mg/g) > corn cobs (77.8 ± 5.9 mg/g) > poplar sawdust (52.7 ± 2.3 mg/g) (Fig. 4b). Figure 4c shows the cellulase activities of the crude protein samples in the rice straw culture. The cellulase and hemicellulase activities increased gradually until peaking at 60 h, and then they decreased. The maximum β-glucosidase, endoglucanase, and exoglucanase activities were 0.33 ± 0.03, 0.19 ± 0.02, and 0.17 ± 0.03 U/mL, respectively (Fig. 4c). Compared to saccharification by *R. thermocellum* M3, the oligosaccharide components produced by a commercial cellulase were the same, and they mainly consisted of glucose, followed by xylose and trace amounts of arabinose (Fig. 4d). The results showed that the oligosaccharides produced by a commercial cellulase gradually increased before 12 h of fermentation and then remained at a constant level (Additional file 8). The compositions of the lignocellulosic feedstock are shown in Additional file 9.

## Discussion

### The cellulosic microorganisms in horse manure

Manure has been recognized as an excellent source of cellulose-fermenting thermophiles [26]. Horse manure is a source of nitrogen, phosphorous, potassium, easily available carbon compounds, microbes, and neutralizing compounds that enable effective growth of the microbes responsible for organic matter decomposition and the consequent rise in temperature [29]. Some typical cellulose-fermenting thermophiles, such as *C. thermocellum* [30], *Clostridium thermobutyricum* [31], *Clostridium thermoaceticum* [32], and *Moorella thermoacetica* [33], have been isolated from horse manure. Erick et al. [27] obtained a cellulosic thermophilic fungus, *A. fumigatus*, which efficiently saccharified cellulose at 45 °C, from horse manure. Wiegel et al. [31] isolated *Clostridium thermobutyricum*, which was able to consume cellulose for butyrate, CO_2_, and hydrogen production, from cellulolytic enrichment cultures that were inoculated with horse manure. In this study, the target functional bacteria were first detected in denaturing gradient gel electrophoresis maps (Additional file 2), indicating that cellulose utilization by the microbial community in the horse manure was stable during the enrichment. In addition, the metabolites that were produced during the enrichment suggested that the horse manure had great potential as a source of cellulosic-saccharifying bacteria (Additional file 1). Eventually, we found that the isolated microorganism *R. thermocellum* M3 consumed cellulose and accumulated oligosaccharides simultaneously, indicating that it has great potential for directly converting cellulose to glucose. Although the strain *R. thermocellum* ATCC 27045 was able to degrade cellulose effectively, little glucose was obtained during the cellulose fermentation. For example, Ellis et al. investigated the metabolic characteristics of *C. thermocellum* ATCC27405 at 60 °C, pH 6.95, and 5 g/L Avicel, and the glucose yield was low [34]. Islam et al. [35] also employed *C. thermocellum* ATCC 27405 to degrade pure α-cellulose fibers of different concentrations at 60 °C, pH 7.3, but no glucose was obtained under low and high α-cellulose concentrations. Tian also found a low glucose yield during the fermentation of sugarcane bagasse (20 g/L) by *C. thermocellum* ATCC 27405 (55 °C, pH 6.99) [36]. However, high glucose accumulation by *R. thermocellum* M3 was detected during the fermentation of cellulose, and glucose was produced preferentially over other saccharification products.

### The accumulation of oligosaccharides in the culture

While the simultaneous growth of microorganisms and the accumulation of oligosaccharides is unlikely, we demonstrated that *R. thermocellum* M3 could accumulate considerable amounts of oligosaccharides during cell growth (Fig. 3c). It was observed that approximately 32 % of the carbon flow was shunted into oligosaccharides after 54 h of fermentation, compared with 18 % at 24 h. This implies that the oligosaccharide concentration continued increasing at low pH, even though bacterial growth ceased. Therefore, we presume that more oligosaccharides were produced than could be consumed during cell growth. As a result, oligosaccharides dramatically accumulated when cell growth was inhibited at pHs below 6.0.

During the batch fermentation of *R. thermocellum* M3, the pH decreased from 7.5 to 6.0 as acetate and lactate accumulated (Fig. 3b). The pH decrease was accompanied by increased oligosaccharide production. It was previously shown that *R. thermocellum* cannot grow well at pHs below 5.8 [37]. However, its cellulolytic enzymes still hydrolyze cellulose under acidic conditions (pH = 4.0–5.5) [38]. The additional glucose utilization also illustrated that low pH limited the further utilization of glucose by microbes while cellulolytic enzymes still retained their activities (Fig. 3b, c; Additional file 6). This phenomenon could potentially explain the high accumulation of fermentable sugars by *R. thermocellum* M3 when cell growth was inhibited. Moreover, the accumulation of fermentable oligosaccharides by *R. thermocellum* M3 was also achieved when using cellulose as the substrate.

### The ratio of glucose to cellobiose

Different from other species of *R. thermocellum*, *R. thermocellum* M3 consumed cellulose without the need for an added β-glucosidase [39], and its maximum β-glucosidase activity was higher than that of some *R. thermocellum* strains [40–42]. In the cellulosome system of *R. thermocellum* M3, the activity of β-glucosidase dominated endoglucanase or exoglucanase activities, which led to a higher oligosaccharide yield and a higher proportion of glucose. The results suggest that the β-glucosidase could hydrolyze most of the cellobiose into glucose.

The extracellular β-glucosidase activity is usually low when *R. thermocellum* degrades cellulose to cellobiose as the main product. It has been reported that the multi-enzymatic system of the cellulolytic bacterium *R. thermocellum* is strongly inhibited by the major-end product cellobiose [43]. Therefore, an additional β-glucosidase usually must be added to *R. thermocellum* cultures if cellulose is to be hydrolyzed efficiently. Fortunately, *R. thermocellum* M3 efficiently hydrolyzed cellobiose to glucose, as was demonstrated by the high specific cellulase activities from both cells and the culture supernatant. Therefore, the high β-glucosidase activity/specific activities could lead to the high proportion of glucose (Fig. 3d; Additional file 7), which was significantly higher than those previously reported for other thermophilic and cellulosic saccharification strains (*P* = 0.009; Table 2).

**Table 2:** Comparison of oligosaccharide yield form various researches with cellulosic substrates

Microorganism	Substrate	Temperature (°C)	Oligosaccharide yield^a^ (mg/g)	References
*Caldicellulosiruptor kristjanssonii*	Microcrystalline cellulose	68	158	[55]
*Ruminiclostridium thermocellum* JN4	Microcrystalline cellulose	60	216	[56]
*Fomitopsis* RCK2010	Wheat straw	50	157	[57]
*Ruminiclostridium thermocellum* S14	Microcrystalline cellulose	60	186	[58]
*Ruminilostridium thermocellum* ATCC27045	Cellobiose	60	18.88	[59]
*Shigella flexner* G3	Microcrystalline cellulose	40	375	[46]
*Ruminiclostridium thermocellum* M3	Microcrystalline cellulose	60	480	This study

^a^The oligosaccharide yield was calculated as oligosaccharides/substrate

## Conclusions

In this study, the novel thermophilic bacterium *R. thermocellum* M3 was isolated from horse manure and characterized. *R. thermocellum* M3 effectively utilized cellulose and raw lignocellulose feedstocks and accumulated oligosaccharides. Different from other strains, *R. thermocellum* M3 exhibited excellent thermophilic and cellulolytic characteristics, as well as great saccharification activity, without the need for pH control or external β-glucosidase addition. In addition, the high proportion of glucose to cellobiose suggests that *R. thermocellum* M3 has great economic potential for the biofuel fermentation industry.

## Methods

### DGGE-PCR

The DNA of the enriched cultures were extracted and purified using the bacterial DNA mini kit (Sango Biotech Co. Ltd., Shanghai, China) according to the manufacturer’s instructions. Denaturing gradient gel electrophoresis (DGGE) was performed according to the methods described by Xing et al. [44]. The variable V3 region of 16S rDNA was enzymatically amplified in the PCR with primers (BSF338 and BSR534) to conserved regions of the 16S rDNA genes [45]. The separation and purification of the PCR products were conducted according to the methods described by Cao et al. [45].

### Enrichment and isolation of the bacteria

The horse manure used as the seed in this research was collected from Mudanjiang farm, Heilongjiang province, China. Enrichment cultivation was conducted in 100-mL top-sealed bottles at 60 °C under static condition, as described by Cao et al. [45]. The cultivation medium was the modified ATCC 1191 (MA) medium mainly composed of 5.0 g/L Avicel, 3 g/L KH_2_PO_4_, 1.5 g/L K_2_HPO_4_**·**12H_2_O, 0.5 g/L (NH_4_)_2_SO_4_, 0.5 g/L NaCl, 0.2 g/L MgSO_4_**·**6H_2_O, 2.0 g/L YE, 0.5 g/L l-cysteine, and 1 mL resazurin (0.2 %). After repeating the enrichment process for five times, tenfold serial dilutions were placed on the solid MA medium (2 %, w/v, agar) prepared in a tube and incubated at 60 °C for 7 days. Agar samples containing well-formed cellulose-clearing colonies were transferred to a fresh MA liquid medium under N_2_ gas flow. The colonies were mashed with a sterilized painting stick to release the cells from the agar. Plating was performed repeatedly to ensure the purity of the isolated colonies. Further verification of purity was ensured by inspecting the microscopy images, colony morphology, and by 16S rDNA gene sequencing.

Genomic DNA of the isolated bacterium was extracted using a bacterial DNA kit (TianGen Biotechnologies Co., Ltd., Beijing, China) and amplified in accordance with the method by Wang [46]. Sequencing was performed at the Sangon Biotechnologies Co., Ltd. (http://www.sangon.com.cn). The nucleotide sequences were compared with those in the GenBank/EMBL/DDBJ nucleotide sequence databases by using the BLAST program (http://www.ncbi.nlm.nih.gov/BLAST/) and the Sequence Match program at the Ribosomal Database Project (RDP). Alignment was conducted using MEGA program (version 6.0). Phylogenetic dendrograms were reconstructed using the MEGA program (version 6.0) [47] with the neighbor-joining (NJ) algorithm and bootstrap analysis of 1000 replicates [48]. The raw sequences data of the strain *R. thermocellum* M3 have been submitted to the NCBI (Accession number: KU695569).

### Fermentation tests

The isolated strain was cultivated anaerobically in MA medium. The inoculum acquired after 48 h incubation was added to 10 % v/v to the fresh MA medium. The cultivation was conducted in 100-mL serum bottles, each bottle containing 50-mL of the MA medium (without nitrogen and carbon source) as described above. The initial pH was adjust by 1 M HCl and 1 M NaOH ranged form 6.0 to 9.0 at intervals of 0.5. The cultivation temperature was increased stepwise from 45 to 70 °C at 5 °C intervals. YE was used as nitrogen source in this research, with 0–2.5 g/L concentration at 0.5 g intervals. Carbon source Avicel was added in 6 different concentrations: 2.0, 3.0, 4.0, 5.0, 6.0, and 7.0 g/L.

### Saccharification of raw lignocellulosic materials under optimal culture conditions

Four different lignocellulosic substrates without pretreatment (except autoclaving at 121 °C for 15 min)—rice straw, corn stalks, corncob, poplar sawdust—were chosen to test *R. thermocellum* M3 saccharification efficiency. The lignocellulosic biomasses were all obtained from Harbin Farm (Harbin, Heilongjiang Province, China). The feedstock was chipped with a Szegvari Attritor System type: B (Union Process Inc.) through a 100-mesh sieve. The experiments were conducted with 5 g/L non-pretreated lignocellulosic materials separately using MA medium as described before. in the cultivation tests. Medium amounting to 100 mL was mixed with 10 mL inocula (the concentration of cell protein was about 280 mg/L), and kept at 60 °C for 72 h. Samples were taken every 6 h to determine oligosaccharide concentration, cell biomass, pH change, as well as cellulose-degraded and liquid-end products.

### Saccharification of raw lignocellulosic materials by commercial cellulase

The commercial cellulase used in this study was purchased from Biotop (Biotopped, China). The saccharification of each raw lignocellulosic materials was performed in the citrate buffer (0.05 mol/L, pH 4.5) with 5.0 g/L lignocellulosic biomass and 2.4 mg/L commercial cellulase under 55 °C. Saccharification samples were taken every 2 h for each 18-h saccharification period to determine the oligosaccharide type and concentration.

### Analytical methods

Oligosaccharides were determined by HPLC (Agilent HP1090) with an Aminex HPX-87H column (Bio-Rad Laboratories, Hercules, CA). Given that the lignocellulosic biomass was insoluble, cell biomass was estimated with the cell protein and determined using the Bradford method [49]. The liquid metabolites were determined by HPLC (4800, Agilent Technologies, USA), as described by Wang et al. [46]. The biogas composition was measured using a gas chromatograph (Agilent Technologies; model 6890N) equipped with a thermal conductivity detector using argon as the carrier gas (capable of detecting concentrations between 200 and 500 ppm). The cellulose concentration was determined in accordance with the method by Huang et al. [50]. The saccharification was calculated as the amount of oligosaccharides produced (mg) per gram of Avicel added. The lignocellulose composition was determined using the automatic cellulose analyzer (ANKOM A200i, USA) in accordance with the manufacturer’s instructions (https://www.ankom.com/product-catalog/ankom-200-fiber-analyzer).

Carbon mass balance was calculated as output carbon mass divided by input carbon mass [46]:

$${\text{Closure}}\left( \% \right) = \left( {{{\left[ {\sum C_{\text{out}} } \right]}{\bigg/}\left[ {\sum C_{\text{in}} } \right]}} \right) \times 100,$$

where ∑*C*_out_ is total carbon recovery in grams and ∑*C*_in_ is initial carbon in grams. Evaluation of the carbon mass balance of cellulosic substrates requires information on initial and final carbon contributions, including cellulose concentrations, cell mass concentrations, soluble protein concentrations, concentrations of sugar, total CO_2_, and organic acids. The whole parameters were measured immediately when inoculation was almost completed and every 6 h thereafter until the end of cultivation (72 h). The carbon contribution from the medium components (primarily YE) was measured with a CHN analyzer (CHNS/O elemental analyzer 2400; Perkin-Elmer, Norwalk, CT). The carbon content of the soluble proteins was estimated to be 50 % (wt/wt) of total protein mass [51]. The concentration of CO_2_ was measured as described by Wang et al. [46].

Cellulase used for saccharification in this research were prepared as the method described by Morag et al. [52]. The cellulase activities were determined by measuring the reducing sugars released from an appropriate substrate in accordance with the method described by Rattanachomsri et al. [53]. The xylanase activities were measured in accordance with the method described by Kohring et al. [54]. One unit of enzyme activity (IU) was defined as the amount of enzyme that produced 1 μmol of reducing sugar per min. All assays were performed in triplicate, and the mean was reported along with the standard deviation.

## Additional files

10.1186/s13068-016-0585-z Oligosaccharides accumulation and fermentative products from Avicel during enrichment cultivation.
10.1186/s13068-016-0585-z DGGE profiles based on 16S rDNA from each enrichment cultivation.
10.1186/s13068-016-0585-z The comparison of 16S rDNA sequences between *R. thermocellum* M3 and similar bacteria species.
10.1186/s13068-016-0585-z The transmission electron microscope (TEM) photograph of *R. thermocellum* M3.
10.1186/s13068-016-0585-z The glucose utilization test by *R. thermocellum* M3 (glucose as a unique substrate.).
10.1186/s13068-016-0585-z The activities analysis of extracellular cellulase.
10.1186/s13068-016-0585-z The specific activities of cellulase from *R. thermocellum* M3.
10.1186/s13068-016-0585-z The oligosaccharide yield of different substrates by commercial cellulase.
10.1186/s13068-016-0585-z The component composition of different lignocellulose feedstocks.

## Abbreviations

Avicel: microcrystalline cellulose
CMC: carboxymethyl cellulose
HPLC: high performance liquid chromatography
MA medium: modified ATCC 1191 medium
RS: rice straw
CS: corn straw
CC: corn cob
PS: poplar sawdust
