# Supplementary material for: Lignocellulosic saccharification by a newly isolated bacterium, Ruminiclostridium thermocellum M3 and cellular cellulase activities for high ratio of glucose to cellobiose
Source: Biotechnol Biofuels. 2016 Aug 11;9:172. doi: 10.1186/s13068-016-0585-z (PMC4982309; doi:10.1186/s13068-016-0585-z)
Supplement: Supplementary file 1 — 10.1186/s13068-016-0585-z Oligosaccharides accumulation and fermentative products from Avicel during enrichment cultivation. [file 13068_2016_585_MOESM1_ESM.docx]

| Enrichment  times | Oligosaccharides  Yield (mg/L) | Lactate  (mg/L) | Ethanol  (mg/L) | Acetate  (mg/L) | Butyrate  (mg/L) | Valerate  (mg/L) |
| --- | --- | --- | --- | --- | --- | --- |
| 1 | 285.00±37.22 | 127.84±32.14 | 26.72±3.87 | 275.93±46.24 | 253.62±51.99 | 17.45±2.94 |
| 2 | 340.00±55.41 | 223.51±18.76 | 27.64±6.77 | 281.41±55.62 | 22.37±4.86 | - |
| 3 | 511.65±32.85 | 245.77±31.32 | 27.81±6.82 | 285.12±34.91 | 20.21±5.75 | - |
| 4 | 884.35±47.84 | 331.24±26.84 | 28.12±3.75 | 291.54±56.66 | - | - |
| 5 | 1329.35±107.88 | 341.82±42.98 | 34.74±9.32 | 312.83±32.24 | - | - |
| 6 | 1595.35±201.34 | 387.26±55.71 | 35.02±8.67 | 336.45±19.86 | - | - |
| 7 | 1932.00±196.25 | 400.21±28.24 | 40.96±2.89 | 346.03±55.28 | - | - |
| 8 | 2265.35±351.21 | 405.85±36.69 | 43.20±10.10 | 557.08±69.02 | - | - |

Additional file 1

**Oligosaccharides accumulation and fermentative products from Avicel during enrichment cultivation**

-: not detected.
