# Supplementary material for: Lignocellulosic saccharification by a newly isolated bacterium, Ruminiclostridium thermocellum M3 and cellular cellulase activities for high ratio of glucose to cellobiose
Source: Biotechnol Biofuels. 2016 Aug 11;9:172. doi: 10.1186/s13068-016-0585-z (PMC4982309; doi:10.1186/s13068-016-0585-z)
Supplement: Supplementary file 2 — 10.1186/s13068-016-0585-z DGGE profiles based on 16S rDNA from each enrichment cultivation. [file 13068_2016_585_MOESM2_ESM.docx]

**Additional file 2**

**DGGE profiles based on 16S rDNA from each enrichment cultivation.**

The DGGE profiles presented the enriched microorganisms in horse manure when using Avicel as substrate. Totally series of enrichment were conducted eight times.
