# Supplementary material for: Lignocellulosic saccharification by a newly isolated bacterium, Ruminiclostridium thermocellum M3 and cellular cellulase activities for high ratio of glucose to cellobiose
Source: Biotechnol Biofuels. 2016 Aug 11;9:172. doi: 10.1186/s13068-016-0585-z (PMC4982309; doi:10.1186/s13068-016-0585-z)
Supplement: Supplementary file 3 — 10.1186/s13068-016-0585-z The comparison of 16S rDNA sequences between R. thermocellum M3 and similar bacteria species. [file 13068_2016_585_MOESM3_ESM.docx]

**Additional file 3**

**The comparison of 16S rDNA sequences between *R. thermocellum* M3 and similar bacteria species.**

Data processing was applied to the results of identify the microbe isolated from horse dung. The contrast [gene sequence](http://www.baidu.com/link?url=VCAGYRVXBVv_ztAOXyyI4B-mzCAYroBRa-D1aKrZ_L9wiLDu8Jq2bTTBTty-NrvfW7OzETFHgYqRfRPH2vKAUd7wOjIuX9D6FqQCGniFIM_AuUO_GQ5Dg1E4IPQWPUlQ)s were obtained from NCBI (<http://www.ncbi.nlm.nih.gov/>). The homology analysis of 16S ribosomal DNA sequences was carried out in software MEGA (version 6.0). The darker of the color, the higher homology of nucleotide sequence.
