# Supplementary figures and images for: Lignocellulosic saccharification by a newly isolated bacterium, Ruminiclostridium thermocellum M3 and cellular cellulase activities for high ratio of glucose to cellobiose
Source: Biotechnol Biofuels. 2016 Aug 11;9:172. doi: 10.1186/s13068-016-0585-z (PMC4982309; doi:10.1186/s13068-016-0585-z)

**Additional file 4**

**The Transmission Electron Microscope (TEM) photograph of *R. thermocellum* M3**


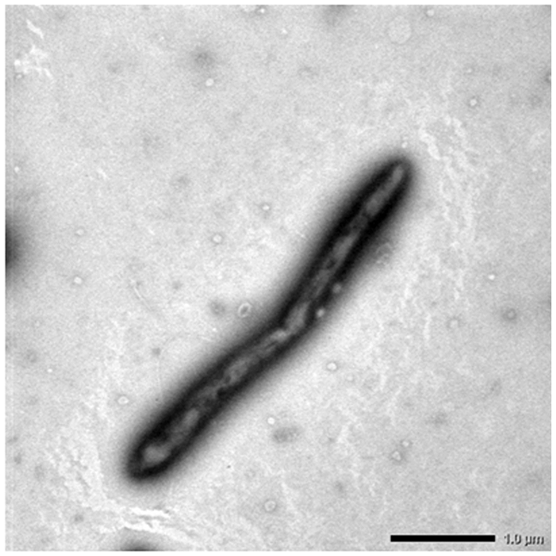

Supplement: Supplementary file 4 — 10.1186/s13068-016-0585-z The transmission electron microscope (TEM) photograph of R. thermocellum M3. [file 13068_2016_585_MOESM4_ESM.docx]
