# Supplementary material for: Lignocellulosic saccharification by a newly isolated bacterium, Ruminiclostridium thermocellum M3 and cellular cellulase activities for high ratio of glucose to cellobiose
Source: Biotechnol Biofuels. 2016 Aug 11;9:172. doi: 10.1186/s13068-016-0585-z (PMC4982309; doi:10.1186/s13068-016-0585-z)
Supplement: Supplementary file 5 — 10.1186/s13068-016-0585-z The glucose utilization test by R. thermocellum M3 (glucose as a unique substrate.). [file 13068_2016_585_MOESM5_ESM.docx]

**Additional file 5**

**The glucose utilization test by *R. thermocellum* M3 (glucose as a unique substrate.).**

The experiment was designed to testify high glucose accumulation under lowing pH by fermentative products. The fermentation was performed in the MA medium (5 g/L Avicel was replaced by 5 g/L glucose as substrate, pH7.5.) under 60 ^o^C. The 10% inoculum (v/v) was added to fresh MA medium using glucose as unique substrate.
