# Supplementary material for: Lignocellulosic saccharification by a newly isolated bacterium, Ruminiclostridium thermocellum M3 and cellular cellulase activities for high ratio of glucose to cellobiose
Source: Biotechnol Biofuels. 2016 Aug 11;9:172. doi: 10.1186/s13068-016-0585-z (PMC4982309; doi:10.1186/s13068-016-0585-z)
Supplement: Supplementary file 7 — 10.1186/s13068-016-0585-z The specific activities of cellulase from R. thermocellum M3. [file 13068_2016_585_MOESM7_ESM.docx]

**Additional file 7**

**The specific activities of cellulase from *R. thermocellum* M3**

The specific activities of cellulase of *R. thermocellum* M3 was calculated as cellulase activity (U/ml)/cell protein (mg/ml). (a) Extracellular specific cellulase activities. (b) Cellular specific cellulase activities.
