# Supplementary material for: Lignocellulosic saccharification by a newly isolated bacterium, Ruminiclostridium thermocellum M3 and cellular cellulase activities for high ratio of glucose to cellobiose
Source: Biotechnol Biofuels. 2016 Aug 11;9:172. doi: 10.1186/s13068-016-0585-z (PMC4982309; doi:10.1186/s13068-016-0585-z)
Supplement: Supplementary file 8 — 10.1186/s13068-016-0585-z The oligosaccharide yield of different substrates by commercial cellulase. [file 13068_2016_585_MOESM8_ESM.docx]

**Additional file 8**

**The oligosaccharide yield of different substrates by commercial cellulase.**
