# Supplementary material for: Lignocellulosic saccharification by a newly isolated bacterium, Ruminiclostridium thermocellum M3 and cellular cellulase activities for high ratio of glucose to cellobiose
Source: Biotechnol Biofuels. 2016 Aug 11;9:172. doi: 10.1186/s13068-016-0585-z (PMC4982309; doi:10.1186/s13068-016-0585-z)
Supplement: Supplementary file 9 — 10.1186/s13068-016-0585-z The component composition of different lignocellulose feedstocks. [file 13068_2016_585_MOESM9_ESM.docx]

**Additional file 9**

**The component composition of different lignocellulose feedstocks.**

|  | Cellulose^a^ | Hemicellulose^a^ | Lignin^a^ | Ash and silicate^a^ |
| --- | --- | --- | --- | --- |
| Rice straw | 50.38±3.32 | 28.72±1.98 | 19.90±2.17 | 1.10±0.03 |
| Corn straw | 44.90±3.31 | 29.52±3.31 | 24.56±2.43 | 1.01±0.02 |
| Corn cob | 40.70±2.58 | 31.80±2.57 | 26.40±1.33 | 1.03±0.02 |
| Poplar sawdust | 23.80±1.89 | 40.88±1.23 | 34.01±2.23 | 1.24±0.05 |

^a^The mean value of lignocellulose composition
